# Cognitive functions and sense of coherence in patients with carotid artery stenosis—Preliminary report

**DOI:** 10.3389/fpsyt.2023.1237130

**Published:** 2023-09-25

**Authors:** Magdalena Piegza, Paweł Dębski, Kamil Bujak, Izabela Jaworska, Piotr Gorczyca, Robert Pudlo, Maciej Żerdziński, Jacek Piegza

**Affiliations:** ^1^Department of Psychiatry, Faculty of Medical Sciences in Zabrze, Medical University of Silesia in Katowice, Tarnowskie Gory, Poland; ^2^Institute of Psychology, Faculty of Social Sciences and Humanities, Humanitas University in Sosnowiec, Sosnowiec, Poland; ^3^Third Department of Cardiology, Faculty of Medical Sciences in Zabrze, Silesian Center for Heart Diseases, Medical University of Silesia in Katowice, Zabrze, Poland; ^4^Department of Cardiac, Vascular and Endovascular Surgery and Transplantology, Silesian Center for Heart Diseases, Medical University of Silesia in Katowice, Zabrze, Poland; ^5^Department of Psychoprophylaxis, Faculty of Medical Sciences in Zabrze, Medical University of Silesia in Katowice, Tarnowskie Gory, Poland; ^6^Faculty of Medicine, Academy of Silesia, Katowice, Poland; ^7^Department of Psychiatry, Dr. Krzysztof Czuma’s Psychiatric Center, Katowice, Poland

**Keywords:** cognitive functions, cognitive impairment, sense of coherence, carotid artery stenosis, carotid artery stenting

## Abstract

**Background:**

There is scarcely any data in the available literature on the relationship between sense of coherence (SOC) and cognitive functioning, and no information on the relationship between SOC and cognitive parameters in patients with carotid atherosclerosis.

**Aim:**

The aim of this paper was to determine the relationship of SOC measured prior to carotid artery stenting (CAS) with neurocognitive functioning in patients with carotid atherosclerosis 12 months after CAS.

**Methods:**

A total of 35 patients with carotid atherosclerosis completed the SOC-29 Orientation to Life Questionnaire (SOC-29) and completed a cognitive test battery twice, i.e., before (baseline–T1) and 12 months after stent implantation (follow-up–T2). Selected cognitive functions such as memory, attention, perception, visuospatial and executive functions and non-verbal fluency were assessed.

**Results:**

One year after the procedure, patients with a higher SOC and sense of manageability present better performance in visual memory. Higher SOC and sense of meaningfulness are positively related to the speed of understanding the changing rules of card sorting (WCST).

**Conclusion:**

Higher overall SOC and a component of sense of meaningfulness and manageability may be related to better cognitive functioning 1 year after the procedure.

## Introduction

There are many scientific reports describing the relationship of sense of coherence (SOC) with quality of life and anxiety, and depression in people with a variety of pathological states (1–8), while few papers address the relationship of SOC with cognitive functioning (9–13).

According to current knowledge, there is no study that explored the interdependencies between SOC and its components and cognitive parameters in people with carotid atherosclerosis. Our study is the first to address this topic and attempts to establish the interdependencies between the aforementioned factors.

Sense of coherence (SOC) consists of 3 components: comprehensibility, manageability and meaningfulness. These are simultaneously cognitive, behavioral and motivational components (14). Comprehensibility conditions the ability to assess reality adequately, manageability denotes the ability to have adequate resources according to the demands of the environment, and meaningfulness–the paramount component, defines the degree of a person’s involvement in his or her own life. All three components of SOC affect each other and, in a sense, may be independent of each other, as there may be situations under which they do not develop to the same degree. The creator of the concept of salutogenesis noted a tendency toward stability in the case of similar levels of all components and a tendency toward equalization of levels when they are strongly differentiated. The process of developing SOC is completed around the age of 30 and thereafter a particular position on the continuum SOC may possibly undergo only minor changes (4, 15). Nevertheless, the change is generally for a high SOC while a low one is likely to become lower and lower over time. In general, SOC is determinant of the essential quality of behavior. It could play a role in the differential response in stressful situations. Antonovsky operated with the concepts of high and low SOC, but failed to specify a cut-off point for low, medium or high SOC (16). Individual researchers were set the cut-off points themselves (17). Some studies suggest that reaching around 140–160 points indicates a strong, 110–130-a moderate, and below 100–a low SOC level (5, 18).

## Aim of the paper

The aim of this paper was to determine the relationship between SOC and neurocognitive functioning in patients with carotid atherosclerosis and, in particular, to explore whether there were a relationship between high SOC measured before carotid artery stenting (CAS) and improvements in cognitive parameters 12 months post-CAS. We endeavored to verify whether the value of SOC was related to the quality of cognitive functioning 1 year after revascularisation.

## Materials and methods

The study involved 35 patients admitted to cardiology departments for carotid artery stenting over a period of three consecutive years (2007–2010). After vascular status had been visualized and the percentage of stenosis had been determined by the Doppler ultrasound, patients were qualified for haemodynamic intervention. Significant stenosis was defined as stenosis of the vessel lumen ≥70% in asymptomatic patients, and in symptomatic patients ≥50% Recovering from ischaemic stroke and/or transient ischaemia (TIA) within the last 6 months served as criteria for symptomatology. Due to the risk of embolisation during the procedure, a distal neuroprotection system in the form of filters was applied to all patients. Participants in the research project performed a cognitive test battery twice, i.e., before (baseline–T1) and 12 months after stent implantation (follow-up–T2). Selected cognitive functions such as memory, attention, perception, visuospatial and executive functions and non-verbal fluency were assessed.

The study was conducted by an experienced clinical psychologist, who assessed and interpreted the results obtained. Inclusion criteria included: age over 18 years, consent to participate in the study, eligibility for carotid artery angioplasty. Patients with severe cognitive impairment were excluded from the study. Due to the small number of symptomatic subjects, two subgroups (symptomatic and asymptomatic) were not distinguished. All patients who successfully completed both phases of the project, i.e., 35 patients, were included in the analysis. They made up one study group.

The Polish adaptation of the Orientation to Life Questionnaire (SOC-29) to assess SOC was used within the study. The adaptation took place in 1993 and is highly reliable. The internal consistency indices are 0.78 for comprehensibility, 0.72 for manageability, 0.68 for meaningfulness and 0.92 for SOC (split-half method with Spearman–Brown correction) (4). Out of 29 questions, 11 refer to comprehensibility, 10 manageability and 8 meaningfulness (15).

The digit symbol test (DS) of the Wechsler Adult Intelligence Scale (WAIS) test was used to estimate non-verbal fluency. This test involves assigning numbers to corresponding symbols. Its completion requires the involvement of cognitive functions such as learning ability, short-term memory, attention concentration and eye-hand coordination. It is a test with time limits (90 s), and the maximum score possible is 93 points. The test score can be transformed into converted scores ranging between 1 and 19 (19).

The Rey–Osterrieth Complex Figure Test (ROCF) is a test that allows the testing of performance in visual memory, visuospatial functions, as well as planning and executive functions. The test consists of the test subject mapping a complex figure and then, after a time interval, redrawing the previously perceived figure from memory. The figure used in the study consists of 18 elements. The level of performance depends on the number of correctly reproduced elements. A maximum of 36 points can be awarded in this test (20).

The Wisconsin card sorting test (WCST) was used to assess executive functions. In this test, the subject is asked to apply an appropriate card sorting rule, based on the tester’s responses limited only to determining whether the rule the subject comes up with is correct. The WCST is therefore a test requiring ability in the area of attention, including organized searching, strategic planning, and the ability to use feedback that is related to information from the subject’s environment. While performing the task, the subject must also demonstrate an ability to modify his or her behavior by suppressing the impulsive application of a single, fixed rule in favor of subordinating his or her reactions to the expectations of the environment (21, 22).

Using a proprietary questionnaire, sociodemographic data and selected clinical parameters were collected and confronted with the data provided in the patient’s discharge.

This study formed part of a broader project, which was approved by the Bioethical Committee of the Silesian Medical University, Ref. No. NN-6501-132/07.

### Statistical analysis

Categorical variables are presented as the number of patients (%). Continuous data are presented as mean (standard deviation; SD) or median (Q1–Q3). The normality of the continuous variables’ distribution was assessed using the Shapiro–Wilk test. The Pearson correlation coefficient or Spearman’s rank correlation coefficient (where appropriate) were utilized to inspect the relationship between SOC and cognitive functions. In addition, the comparisons of SOC-total values between patients stratified by baseline characteristics and the change of cognitive function parameters (between examinations at baseline and 12 months after) according to dichotomized SOC components (by median) were performed using *t*-test. The level of statistical significance was *p* < 0.05 (two-tailed). All statistical analyses were performed using Statistica version 13.3 (TIBCO Software, CA, USA).

## Results

Ultimately, 35 people participated in the study, aged between 58 and 68 years, 34.3% of whom were women. The symptomatology criterion was met by 20% of the subjects. Sociodemographic characteristics and selected clinical parameters of the described group are presented in the Table 1.

**TABLE 1:** Sociodemographic and clinical characteristics.

Characteristics	All patients (*n* = 35)
Sex (female)	12 (34.3%)
Age (years)	65 (58–68)
**Level of education**	
Primary	7 (20.6%)
Vocational	4 (11.8%)
Secondary	15 (44.1%)
Higher	8 (23.5%)
**Employment**	
Professionally active	3 (8.6%)
Pensioner or unemployed	32 (91.4%)
**Marital status**	
Single or divorced or widowed	8 (22.9%)
Married	27 (77.1%)
Mental disorders	2 (5.7%)
Previous stroke or TIA	7 (20.0%)
Previous CAS	1 (3.0%)
Previous CAD	31 (88.6%)
Previous PCI or CABG	18 (51.4%)
Malignancy	3 (8.6%)

Categorical variables are shown as a number of patients (percentage). Quantitative variables are presented as median (Q1–Q3).

TIA, transient ischemic attack; CAS, carotid artery stenting; CAD, coronary artery disease; PCI, percutaneous coronary interventions; CABG, coronary artery bypass grafting.

Patients completed the SOC questionnaire (SOC-29) before carotid angioplasty. Cognitive functioning was assessed before and 12 months post CAS. However, the next stage of the study was completed only by those who, after a telephone invitation, attended a follow-up visit, maintained a consent to the study and completed the cognitive tests in full. The said accounted for a total of 25 people.

On the second measurement (T2), those with a higher baseline sense of meaningfulness needed fewer trials to understand the card sorting rule on the Wisconsin card sorting test (TC1sc–trials to complete the first category, *p* = 0.04). A similar relationship was observed between TC1sc and overall SOC (*p* = 0.03). A positive correlation was obtained between manageability and SOC-TOTAL and Rey–Osterrieth Complex Figure–delayed recall (ROCF-dr, *p* = 0.01, *p* = 0.02, respectively). These relationships were statistically significant. The results obtained in the second measure are shown in the Table 2.

**TABLE 2:** Correlations between SOC and cognitive functions in follow-up (T2) (*n* = 25).

	MEA	MAN	COM	SOC-TOTAL
%E	0.09	0.20	0.16	0.19
%NPE	−0.07^s^	−0.08^s^	−0.12^s^	−0.14^s^
%PE	−0.15^s^	−0.15^s^	−0.31^s^	−0.24^s^
%PE-C	0.18	0.28	0.34	0.33
%PE-SR	−0.30	−0.34	−0.40	−0.42
%PR	0.08^s^	0.08^s^	0.19^s^	0.14^s^
%CLR	−0.22^s^	−0.11^s^	−0.20^s^	−0.18^s^
%CLR-C	−0.13	−0.18	−0.21	−0.22
%CLR-SR	−0.24	−0.24	−0.25	−0.29
NPE	−0.04^s^	0.07^s^	0.06^s^	0.10^s^
NPE-C	−0.10^s^	−0.13^s^	−0.10^s^	−0.15^s^
NPE-SR	−0.10	−0.02	0.01	−0.03
PE	−0.10^s^	−0.08^s^	−0.24^s^	−0.16^s^
PE-C	−0.02^s^	0.01^s^	0.18^s^	0.09^s^
PE-SR	−0.10^s^	−0.08^s^	−0.24^s^	−0.16^s^
TNE	−0.15^s^	0.00^s^	−0.10^s^	−0.06^s^
TNE-C	0.11	0.15	0.18	0.18
TNE-SR	−0.28	−0.21	−0.21	−0.26
NCS	−0.18	−0.22	−0.06	−0.17
NTP	0.02^s^	−0.10^s^	0.08^s^	0.00^s^
NCC	−0.05^s^	−0.02^s^	−0.22^s^	−0.11^s^
PR	0.01^s^	0.02^s^	0.14^s^	0.07^s^
PR-SR	−0.02^s^	−0.18^s^	−0.15^s^	−0.12^s^
PR-C	−0.38	−0.09	−0.31	−0.29
CLR	−0.19	−0.26	−0.17	−0.24
TC1sc	−**0.42^s,d^**	−0.40^s^	−0.35^s^	−**0.44^s,c^**
FMS	−0.23^s^	−0.27^s^	−0.15^s^	−0.19^s^
DS	0.11	0.11	−0.17	−0.01
DS-c	−0.01^s^	0.15^s^	−0.15^s^	−0.01^s^
ROCF-c	0.09^s^	−0.11^s^	−0.21^s^	−0.13^s^
ROCF-dr	0.37	**0.53^a^**	0.37	**0.49^b^**

^a^*P* = 0.01. ^b^*P* = 0.02. ^c^*P* = 0.03. ^d^*P* = 0.04. ^s^Spearman’s rank correlation coefficient.

Bold text represents statistically significant correlations.

MEA, meaningfulness; MAN, manageability; COM, comprehensibility; SOC-TOTAL, sense of coherence total score; %E, percentage of errors; %NPE, percentage of non-perseverative errors; %PE, percentage of perseverative errors; %PE-C, percentage of perseverative errors-centile; %PE-SR, percentage of perseverative errors-standardized results; %PR, percentage of perseverative responses; %CLR, percentage of conceptual level responses; %CLR-C, percentage of conceptual level responses-centile; %CLR-SR, percentage of conceptual level responses-standardized results; NPE, non-perseverative errors; NPE-C, non-perseverative errors-centile; NPE-SR, non-perseverative errors-standardized results; PE, perseverative errors; PE-C, perseverative errors-centile; PE-SR, perseverative errors-standardized results; TNE, total number errors; TNE-C, total number errors-centile; TNE-SR, total number errors-standardized results; NCS, number of correct sorts; NTP, number of tests performer; NCC, number of categories completed; PR, perseverative responses; PR-SR, perseverative responses-standardized results; PR-C, perseverative responses-centile; CLR, conceptual level responses; TC1sc, trials to complete the first category; FMS, failure to maintain set; DS, digit symbol test; DS-c, digit symbol test-convert; ROCF-c, Rey–Osterrieth Complex Figure-copy; ROCF-dr, Rey–Osterrieth Complex Figure-delayed recall.

No differences in SOC-TOTAL were observed in the study group taking into account the age of the subjects, gender, marital status and symptomatology. SOC was not compared in relation to education and employment due to the impossibility of dichotomising these parameters. The relationships discussed are illustrated in the Table 3.

**TABLE 3:** The comparison of the mean SOC-TOTAL between patients stratified by age, sex, marital status, employment and presence of symptoms of carotid artery stenosis.

	Group 1	Group 2	*P*-value
Age < 65 (*n* = 18) vs. > 65 years (*n* = 17)	139.3 (29.9)	151.5 (20.2)	0.17
Men (*n* = 23) vs. women (*n* = 12)	139.4 (23.3)	156.5 (28.4)	0.06
Married (*n* = 27) vs. single or divorced or widowed (*n* = 8)	142.6 (26.3)	154.1 (24.5)	0.28
Pensioner or unemployed (*n* = 32) vs. professionally active (*n* = 3)	144.8 (25.0)	150.3 (42.2)	0.73
Symptomatic (*n* = 13) vs. asymptomatic (*n* = 22)	142.7 (26.7)	146.8 (26.2)	0.66

Values are presented as mean (standard deviation).

Considering the assessment of the severity of SOC within its three components (using the median as a cut-off point for higher and lower severity), better cognitive ability regarding visual memory (ROCF-dr) was observed 12 months upon the procedure in subjects with higher scores on sense of manageability (Table 4).

**TABLE 4:** Cognitive performance depending on the level of psychological coherence.

Absolute change from baseline	COM ≤ median	COM > median	*P*-value	MAN ≤ median	MAN > median	*P*-value	MEA ≤ median	MEA > median	*P*-value	SOC ≤ median	SOC > median	*P*-value
ROCF-c	1.3 (4.6)	3.7 (5.9)	0.26	3.4 (5.9)	1.3 (4.3)	0.31	2.6 (5.1)	2.1 (5.6)	0.81	1.5 (4.8)	3.4 (5.7)	0.37
ROCF-dr	1.9 (5.3)	3.5 (4.0)	0.43	0.6 (5.0)	4.7 (3.5)	0.03	1.0 (5.0)	4.3 (4.0)	0.10	1.3 (5.5)	4.0 (3.5)	0.19
DS	0.5 (10.6)	3.2 (7.1)	0.49	−1.2 (11.6)	4.7 (4.1)	0.13	0.7 (11.0)	2.7 (7.0)	0.60	0.2 (11.0)	3.3 (6.7)	0.43
DS-c	0.1 (3.3)	0.6 (2.3)	0.67	−0.6 (3.7)	1.3 (1.0)	0.12	0.0 (3.4)	0.6 (2.2)	0.60	−0.1 (3.4)	0.7 (2.1)	0.51
NTP	−7.6 (18.2)	−5.7 (22.7)	0.82	−5.8 (16.0)	−7.8 (24.1)	0.81	−6.6 (20.6)	−7.0 (20.0)	0.96	−8.8 (18.3)	−4.6 (22.0)	0.60
NCS	1.1 (11.9)	3.4 (11.8)	0.64	4.5 (11.0)	−0.5 (12.2)	0.30	0.5 (10.7)	3.8 (12.9)	0.48	1.4 (12.2)	2.8 (11.6)	0.76
TNE	−8.0 (14.0)	−9.0 (20.0)	0.88	−9.5 (13.0)	−7.3 (20.2)	0.74	−6.3 (13.2)	−10.8 (19.9)	0.51	−9.5 (13.7)	−7.3 (19.7)	0.76
TNE-SR	5.9 (11.3)	6.0 (11.7)	0.98	6.8 (11.0)	5.0 (11.9)	0.70	5.1 (11.2)	6.8 (11.7)	0.70	6.8 (11.3)	5.0 (11.6)	0.70
TNE-C	10.6 (20.4)	11.5 (24.1)	0.93	13.9 (20.0)	7.8 (23.8)	0.49	8.8 (18.5)	13.4 (25.2)	0.60	12.4 (20.2)	9.5 (24.0)	0.75
PR	−6.7 (12.6)	−7.3 (13.8)	0.92	−7.6 (12.2)	−6.3 (14.1)	0.80	−4.6 (8.4)	−9.5 (16.5)	0.35	−8.5 (12.0)	−5.3 (14.1)	0.54
PR-SR	7.2 (13.6)	4.1 (20.5)	0.65	5.1 (19.0)	6.7 (14.5)	0.82	7.2 (12.9)	4.3 (20.4)	0.67	9.9 (13.1)	1.4 (19.5)	0.21
PR-C	11.6 (22.6)	18.0 (30.2)	0.55	17.0 (22.8)	11.6 (29.6)	0.61	9.5 (18.7)	19.8 (31.9)	0.33	15.4 (20.7)	13.3 (31.4)	0.85
%PR	−4.7 (9.9)	−5.6 (9.3)	0.81	−5.7 (9.7)	−4.5 (9.7)	0.76	−3.5 (6.6)	−6.8 (11.9)	0.40	−6.4 (9.5)	−3.8 (9.6)	0.50
PE	−5.9 (11.5)	−5.5 (11.8)	0.92	−6.0 (11.6)	−5.4 (11.7)	0.90	−3.5 (7.7)	−8.2 (14.3)	0.31	−7.5 (10.9)	−3.8 (12.0)	0.44
PE-SR	7.9 (14.4)	7.5 (13.3)	0.96	8.1 (14.5)	7.3 (13.3)	0.89	7.2 (14.1)	8.3 (13.7)	0.83	10.5 (14.1)	4.8 (13.0)	0.30
PE-C	12.4 (24.3)	12.5 (26.9)	0.99	12.8 (25.3)	12.1 (25.7)	0.95	8.0 (20.9)	17.3 (28.9)	0.37	15.5 (23.0)	9.1 (27.6)	0.53
%PE	−4.4 (9.0)	−4.3 (7.6)	0.97	−4.6 (9.1)	−4.0 (7.5)	0.85	−2.9 (6.0)	−5.9 (10.2)	0.37	−5.7 (8.6)	−2.8 (7.8)	0.39
%PE-SR	8.0 (14.5)	7.5 (13.0)	0.94	8.6 (14.9)	6.9 (12.7)	0.76	7.2 (13.6)	8.5 (14.2)	0.81	10.8 (14.0)	4.5 (12.9)	0.25
%PE-C	13.0 (25.8)	13.5 (27.2)	0.96	13.7 (26.9)	12.8 (26.0)	0.93	8.9 (21.4)	17.9 (30.3)	0.40	17.0 (24.0)	9.2 (28.3)	0.46
NPE	−2.6 (7.9)	−11.1 (30.1)	0.32	−9.9 (26.7)	−2.5 (11.2)	0.38	−2.9 (9.0)	−10.1 (28.6)	0.40	−2.0 (8.2)	−11.1 (28.6)	0.28
NPE-SR	3.4 (9.1)	7.6 (20.7)	0.50	7.2 (17.6)	3.2 (12.1)	0.51	3.2 (10.3)	7.6 (19.2)	0.47	2.4 (9.5)	8.4 (19.4)	0.33
NPE-C	6.5 (19.8)	8.1 (29.4)	0.87	8.6 (23.3)	5.8 (25.0)	0.78	8.5 (21.9)	5.5 (26.6)	0.76	5.7 (20.7)	8.9 (27.7)	0.75
%NPE	−1.8 (5.7)	−1.0 (9.6)	0.81	−2.4 (6.6)	−0.3 (8.5)	0.49	−1.8 (6.5)	−1.0 (8.7)	0.79	−1.1 (5.8)	−1.8 (9.3)	0.84
CLR	3.9 (13.1)	3.0 (15.1)	0.87	8.2 (12.3)	−1.6 (13.8)	0.07	2.4 (8.5)	4.8 (18.1)	0.68	5.0 (13.1)	1.9 (14.7)	0.59
%CLR	8.2 (13.8)	6.1 (16.7)	0.73	10.4 (12.9)	3.9 (16.6)	0.29	6.7 (12.4)	7.8 (17.6)	0.86	10.1 (13.2)	4.3 (16.5)	0.34
%CLR-SR	6.6 (12.0)	5.6 (12.5)	0.85	8.5 (11.0)	3.6 (12.9)	0.31	6.5 (11.6)	5.8 (12.9)	0.90	8.8 (11.7)	3.3 (12.1)	0.25
%CLR-C	11.2 (22.4)	8.4 (24.9)	0.77	15.7 (21.0)	3.8 (24.5)	0.20	9.2 (18.8)	10.8 (27.9)	0.87	14.0 (21.3)	5.6 (25.1)	0.37
NCC	0.5 (1.6)	0.0 (1.5)	0.42	0.5 (1.6)	0.0 (1.4)	0.39	0.1 (1.1)	0.5 (1.9)	0.50	0.5 (1.6)	0.0 (1.4)	0.39
TC1sc	117.5 (29.9)	128.9 (8.7)	0.15	116.1 (31.0)	129.7 (5.8)	0.08	122.6 (16.7)	123.2 (29.1)	0.94	117.1 (30.8)	128.6 (8.5)	0.15
FMS	0.3 (1.8)	0.5 (2.5)	0.76	0.8 (2.0)	−0.1 (2.1)	0.27	0.6 (1.9)	0.2 (2.3)	0.60	0.3 (1.9)	0.5 (2.4)	0.82

Value are presented as mean (standard deviation).

MEA, meaningfulness; MAN, manageability; COM, comprehensibility; SOC, sense of coherence total score; %NPE, percentage of non-perseverative errors; %PE, percentage of perseverative errors; %PE-C, percentage of perseverative errors-centile; %PE-SR, percentage of perseverative errors-standardized results; %PR, percentage of perseverative responses; %CLR, percentage of conceptual level responses; %CLR-C, percentage of conceptual level responses-centile; %CLR-SR, percentage of conceptual level responses-standardized results; NPE, non-perseverative errors; NPE-C, non-perseverative errors-centile; NPE-SR, non-perseverative errors-standardized results; PE, perseverative errors; PE-C, perseverative errors-centile; PE-SR, perseverative errors-standardized results; TNE, total number errors; TNE-C, total number errors-centile; TNE-SR, total number errors-standardized results; NCS, number of correct sorts; NTP, number of tests performer; NCC, number of categories completed; PR, perseverative responses; PR-SR, perseverative responses-standardized results; PR-C, perseverative responses-centile; CLR, conceptual level responses; TC1sc, trials to complete the first category; FMS, failure to maintain set; DS, digit symbol test; DS-c, digit symbol test-convert; ROCF-c, Rey–Osterrieth Complex Figure-copy; ROCF-dr, Rey–Osterrieth Complex Figure-delayed recall.

## Discussion

The results of a previously published study showed improvements in the following cognitive functions: psychomotor speed, visuospatial episodic memory and executive function in patients 1 year after CAS. Importantly, we failed to observe a decline in any of the analyzed cognitive abilities after CAS at 1-year follow-up (23). Similar conclusions are also reached by other researchers (24–31), who take the position that carotid artery stenting improves cognitive functioning in both basic and more complex cognitive functions in people with carotid atherosclerosis.

Atherosclerosis in the carotid arteries is associated with the risk of ischemic stroke and cognitive deterioration, primarily from embolization and chronic hypoperfusion of brain tissue. Revascularization procedures result in improved cerebral hemodynamics and reduced embolization, which in some patients, in addition to stroke protection, can lead to improvement in cognitive functioning. Both factors related to the medical procedure and those dependent on the patient are responsible for the global neurocognitive effect after carotid revascularization. Transient neurocognitive deficits may occur during CAS, which are prevented by the neuroprotection systems used during the procedure itself (32). A close relationship between improved cerebral blood supply and favorable changes in cognitive tests has been noted by many authors (28, 33, 34), and some believe that impaired cerebral blood supply may be an independent determinant of cognitive degradation in patients with severe stenosis (35). Hence, the creators of the randomized CREST-H trial proposed supplementing the concept of “symptomatic carotid artery stenosis” with cognitive deterioration, which they believe could be included alongside ischemic cerebral stroke, transient ischemic attacks (TIA) and amaurosis fugax in the symptom criterion (36). More recent data address the relationship between compensatory functional connectivity (FC) changes in specific brain structures and cognitive parameters. Significant FC abnormalities are associated with poorer memory and executive functions, and have shown a tendency to improve after stenting (37).

The Canadian researchers described 2 cases of patients with carotid stenosis in whom they observed an improvement in cognitive parameters concerning the area supplied by the artery being stented 2 months after CAS. The improvement was more pronounced in the patient with more severe stenosis (> 95% in the right carotid artery) compared to the patient with 70% stenosis in the left internal carotid artery. Using task-phase functional magnetic resonance imaging (fMRI), the activation of specific brain areas was checked during a battery of computer-assisted cognitive tests. The first case the study showed an increased activation on the right (treated) side of the frontal and temporal lobes 2 months after stenting associated with an increase in accuracy and task completion rate (24). The results of the cited study are in the mainstream supporting the hypothesis of improved cognitive function after CAS. The same researchers also highlight the role of fMRI in recording functional changes in the specific brain areas, which are strongly correlated with neurocognitive improvement after revascularisation, as assessed by the standard tools (25).

According to the state of art, there are no studies describing the interdependencies between SOC and its components and specific cognitive exponents. Our study is the first to attempt to establish these relationships in relation to cognitive functions such as psychomotor speed, visuospatial episodic memory and executive function in patients with carotid artery stenosis who undergone CAS. Furthermore, only a few articles were found describing lower cognitive functioning in people with specific disorders and lower SOC (1, 9, 12, 13).

Our study revealed that the higher the sense of manageability and the overall SOC, the higher the visual memory performance 12 months after the procedure with regard to the task of reproducing cognitive material after a time delay (ROCF-dr). No correlations were observed regarding non-verbal fluency (digit symbol test) and components of SOC. Moreover, people with a higher sense of meaningfulness understand the rules of card sorting more quickly 1 year after revascularisation.

In other studies, a high level of SOC was significantly correlated with the self-reported quality of cognitive functioning (10). Given the obvious impact of emotional functioning on general health condition, no association was found between SOC and the physical aspect of health, but strong correlations with its mental aspect were confirmed (2). The salutogenic effect of a high SOC, as well as its relationship with lower parameters of depression and anxiety, in line with theoretical assumptions, was perceived by other authors (4–6).

In a cross-sectional study of a Norwegian population of 975 people in rehabilitation with musculoskeletal, cardiovascular, neurological conditions, neoplasms and other diseases, to determine the relationship of SOC with disability and quality of life, it was shown that a high level of SOC is linked to lower disability in mental domains especially in patients with cardiovascular diseases. It should be noted that this project used the World Health Organization Disability Assessment Schedule 2.0 (WHODAS 2.0) to estimate disability, which devotes one domain to cognitive functions (9).

Similarly, in an earlier Norwegian study, a higher level of SOC had been associated with higher quality of life in people ≥ 65 years of age without any cognitive impairment during hospitalization (general public hospital), while no such relationship had been observed after 12 months. It is worth highlighting that the most common diagnosis due to which patients had been hospitalized proved to be cardiovascular and/or respiratory diseases (1).

A low SOC was shown in adults with FAS (Fetal Alcohol Syndrome) compared to a control group of healthy subjects (for FAS the median of the SOC score amounted to 112, for the control group–133). Neurocognitive deficits were also reported in the described patient group (cognitive and executive functions and social cognition) (12).

Deficits in cognitive and emotional functioning were described in people with autism spectrum disorder (ASD), which can be remediated by enhancing SOC, according to the salutogenetic concept of understanding health and physical and mental wellbeing (13).

Subsequent studies focus on the analysis of overall functioning, quality of life, life satisfaction, adaptation to chronic disease and also mortality in relation to the strength of SOC (3, 7, 8, 38–45).

The Belgian researchers examined 567 primary care patients aged ≥ 80 years with multiple chronic diseases for the relationship of SOC to mortality and first non-planned hospitalization and impairment of activities of daily living (ADLs) during a 1.5-year and 3-year follow-up. They concluded that even older people with high SOC are characterized by longer survival and less impairment of activities of daily living despite the coexistence of multiple life-shortening diseases. This protective effect of high SOC was shown to be independent of multimorbidity, presence of depression, level of cognitive functioning, degree of disability and demographics (3). Similarly, results from the CAIDE Study (Cardiovascular Risk Factors, Aging and Dementia Study) report a link between high anxiety and increased risk of MCI/dementia and death. A high score on the 13-item SOC scale was associated with lower mortality. The presence of depressive symptoms attenuated these relationships (38). These studies demonstrate a protective effect of SOC extending beyond sense of health (perceived health) and quality of life to include mortality and functional impairment even for populations predisposed to adverse health outcomes (3, 38).

Data sourced from various centers report that strong SOC is related to lower disability (3, 39, 46).

Sense of coherence (SOC) of the spouse of a stroke survivor was significantly associated with life satisfaction, satisfaction with relationship, sex life, financial situation before the stroke, and positive anticipation of the future, self-control, general health, and vitality at the time of the study. In contrast to other reports, there was no strong relationship of SOC with the severity of anxiety and depressive mood in this study, nor with the severity of the impairments of the stroke victims resulting from the stroke in the person being cared for by the subject (44).

Sense of coherence (SOC) was proven to be a predictor of the overall outcome of tasks involving cognitive ability to an equal extent in men and women. People with higher SOC subjected to induced stress performed better on tasks compared to those with lower SOC, whereas no analogous relationship was observed in the no-stress group (45).

In adults with multiple somatic disorders, SOC was shown to be a determinant of quality of life, particularly related to its psychological component (7). Other researchers also described a significant positive relationship between SOC and quality of life in other chronic patients, such as those on haemodialysis (8). In women diagnosed with breast cancer, high SOC was found to be a psychological factor playing a protective role in adaptation to neoplastic disease (40). Whereas those with a strong SOC, complaining of chronic pain in the course of various somatic diseases exhibited more mature coping mechanisms and were less prone to catastrophic thinking. Prayer and sustaining hope appeared to be the most common coping methods, the least frequent methods used by the study participants were methods based on changing their understanding of the substance of pain (47). Moreover, the researchers emphasize the role of education in pain management in chronically ill, older adults (46). In contrast to the previous study, older adults ≥ 65 years without any impairment of cognitive functions had improved the SOC parameters 1 year after the end of hospitalization for somatic reasons, with a higher score on the SOC Scale being linked to lower SOC on the first measure (during their hospitalization) and not receiving help from others for the duration of the project. It is noteworthy that a total of 97 participants took part in the study, with a valid score on the mini mental state examination (MMSE) (47). Both patients after their first stroke with current neurological symptoms but any without significant cognitive deterioration and their caregivers with low SOC presented more difficulties in coping with their life situation compared to those with strong SOC within a period of 3 months after discharge (47).

The orientation to life model developed by Antonovsky can be used to understand the life satisfaction of older people (2). The Support Monitoring and Reminder Technology for Mild Dementia (SMART4MD) project investigated how older people with cognitive impairment manage health programmes with the use of mobile applications. Data analysis was conducted based on the theoretical assumptions of the SOC model for all three of its components. Although the results of the study were identified as ambiguous, SOC proved to be useful in gaining a deeper understanding of the motives for engaging in specific activities related to the use of new technologies (42).

The application of the concept of salutogenesis in health promotion and work on enhancing SOC in different populations of people to improve health using individual resources also received considerable consideration in the literature (2, 10, 11, 14, 48). Salutogenesis is a promising concept for use in health promotion for older, vulnerable people with cognitive deterioration (14).

Within the studies of the Finnish population, good cognitive functioning, physical activity and being married for men were linked to higher SOC, which also correlated positively in particular with psychological and social determinants of health. The authors of the study quoted above sought to answer the question of how generalized resistance resources, consisting of income, cognitive functioning, a number of years of education, a marital status and physical activity affect SOC and state of health (physical, social and mental health) (11).

For the purpose of identifying specific methods of strategies to enhance SOC in patients with dementia, 3 areas of nursing activities were identified on the basis of observations and interviews: seeking individual resources, adjusting the type of activity and finding creative solutions. These activities are intended to improve and enhance the quality of care for people with dementia (48). In another study, researchers in Taiwan demonstrated a beneficial effect of horticultural therapy applied for 12 weeks but in people without dementia requiring long-term care on SOC levels (49). The positive role of SOC in health promotion is also emphasized by other authors, while postulating the need for further research (10, 14).

The human brain could adapt to changing living conditions throughout life because of specific neuronal and network brain mechanisms that reshape brain functions. The basis of brain development was neuroplasticity which consisted of structural and functional brain reorganizations such as changes in white matter myelination, gray matter volume, creation of novel synaptic connections, and reconstruction of already existing synapses (50). Plasticity could be stimulated by cognitively engaging activities: reading, learning to play a musical instrument, physical exercise, studies, etc (50). Exercise was very important for brain rehabilitation and could improve cognitive function by modulating molecular and cellular mechanisms within the brain. Also changes in the brain under the influence of cognitive rehabilitation were caused by the plasticity of the brain. Cognitive training modified brain networks and brain structural changes, what was connected with reorganizing existing synaptic connections. Rehabilitation of cognitive functions was often used in elderly people, especially with mild cognitive impairment (MCI). Carotid atherosclerosis was considered as an independent risk factor for the transformation process of MCI to dementia (33). There was a high probability that this population of patients would benefit from cognitive rehabilitation. To stimulate new formation of more adaptative circuits to achieve cognitive benefits–e.g., neurofeedback therapy, musical training, computerized cognitive training programs, abacus training, aerobic and resistance training, brain-computer interface technology, transcranial non-invasive brain stimulation (NIBS), and Strategic Memory Advanced Reasoning Training (SMART) could be used (50–53). Some researchers have proposed combining cognitive training with pro-cognitive medication as some alternative opportunity to modify brain networks (54).

### Limitations of the study

The main limitation of the paper is the small number of people participating in the study, due to objective difficulties in obtaining the research material. This further decreased in the second measurement, which also been contributed to by the mortality of several subjects. Accessibility to the patient with carotid atherosclerosis may be limited due to the nature of the disease, potential problems in maintaining sufficient striving tension to complete the study, surviving to the end of a comprehensive cognitive function test and completing the same cognitive test battery 1 year after the procedure. It is worth noting that the available literature rarely reports a larger number of patients participating in the similar projects. In our study, men often suffering from other medical conditions and with generalized atherosclerosis predominate. We were focusing on examining the relationship between SOC and only selected domains of cognitive functioning. For comparisons of variables considering sociodemographic data, only those data were qualified where a dichotomous division was possible. It would be worth expanding both the study group and the study methodology in the future, taking advantage of the ever-increasing possibilities in this regard.

## Conclusion

(1) Higher SOC and sense of manageability are associated with better visual memory ability 1 year after stenting in patients undergoing revascularisation of the carotid arteries.
(2) People with higher SOC, including meaningfulness, understand the changing rules of card sorting (WCST) more quickly, which may mean that they present more efficient executive functions 1 year after the procedure.

## Data availability statement

The original contributions presented in this study are included in the article/supplementary material, further inquiries can be directed to the corresponding author.

## Ethics statement

The studies involving humans were approved by the Bioethical Committee of the Medical University of Silesia in Katowice, Poland. The studies were conducted in accordance with the local legislation and institutional requirements. The participants provided their written informed consent to participate in this study.

## Author contributions

MP and JP: conceptualization, methodology, and writing-original draft preparation. MP, PD, and IJ: validation. MP, PD, RP, and PG: formal analysis. MP, JP, and IJ: investigation. MP, KB, and PD: resources and data curation. MŻ: writing-review and editing. PD and MP: visualization. PG, RP, and MŻ: supervision. MP and KB: project administration. All authors have read and agreed to the published version of the manuscript.
